# Type 2 diabetes mellitus: A central nervous system etiology

**DOI:** 10.4103/2152-7806.66460

**Published:** 2010-07-16

**Authors:** Peter J. Jannetta, Lynn H. Fletcher, Peter M. Grondziowski, Kenneth F. Casey, Raymond F. Sekula Jr

**Affiliations:** Department of Neurosurgery, Allegheny General Hospital, 420 East North Avenue, Suite 302; 1 Department of Neurosurgery, Allegheny General Hospital, Pittsburgh, PA USA 15212; 2 Center for Diabetes and Endocrine Health, Allegheny General Hospital, Pittsburgh, PA USA 15212; 3 Oakwood Southshore Hospital, Department of Neurosurgery, Trenton, MI USA 48201

**Keywords:** Body mass index, diabetes mellitus, lateral medullary, microvascular decompression, type 2 diabetes, vascular cross-compression

## Abstract

**Background:**

Insulin resistance (hyperinsulinemia) is said to be the signal event and causal in the development of type 2 diabetes mellitus. Pulsatile arterial compression of the right anterolateral medulla oblongata is associated with autonomic dysfunction, including “driving” the pancreas, which increases insulin resistance causing type 2 diabetes mellitus. In this prospective study, we hypothesize that decompressing the right cranial nerve X and medulla will result in better glycemic control in patients with type 2 diabetes mellitus.

**Methods:**

Ten patients underwent retromastoid craniectomy with microvascular decompression for type 2 diabetes mellitus. Patients were followed for 12 months postoperatively by blood glucose monitoring and studies of glycemic control, pancreatic function and insulin metabolism. No changes in diet, weight or activity level were permitted during the course of the project.

**Results:**

Seven of the 10 patients who received microvascular decompression for type 2 diabetes mellitus showed significant improvement in their glucose control. This was noted by measurement of diabetes markers and decrease of diabetes medication dosages. One patient was completely off diabetes medication, while attaining euglucemia. The other 3 patients did not improve in their glucose control. The body mass index of these 3 patients was higher (mean, 34.4) than those with better outcomes (mean, 27.9).

**Conclusion:**

Arterial compression of the right anterolateral medulla appears to be a factor in the etiology of type 2 diabetes mellitus. Microvascular decompression may be an effective treatment for non-obese type 2 diabetes mellitus patients.

## INTRODUCTION

Over the past 43 years, we have studied pulsatile vascular compression of the cranial nerves. We demonstrated that a number of hyperactive dysfunction syndromes were not only caused by vascular compression but that they could be relieved without loss of function by mobilizing the offending blood vessel or vessels away from the nerve (microvascular decompression, MVD).[1,2] It is now generally accepted in the international neurosurgical community that vascular cross-compression causes cranial nerve hyperactive syndromes. These include, among others, trigeminal neuralgia, hemifacial spasm, vertigo and disequilibrium, Miniere‘s disease, glossopharyngeal neuralgia and spasmodic torticollis.[2–18] In 1973 the first observations were made on essential hypertension, which proved to be a vascular compression syndrome of the left anterolateral medulla.[19] The vagus nerve is the only cranial nerve with an asymmetrical distribution. The first MVDs were performed for essential hypertension in 1975 and reported in 1979. More definitive papers followed in 1985 and later.[20–23] A subhuman primate model of essential hypertension was developed.[24] This work is well known and has been verified by others.[25–27]

In this series, we found that 86% of the patients could be improved or relieved of their hypertension by microvascular decompression of the left lateral medulla; half of the patients were able to discontinue all antihypertensives; and in the other half, the medication dosage was decreased significantly. This work has been verified by a number of investigators.[27–29] On the basis of the above, it became reasonable to assume that there should be a right lateral medullary syndrome The pancreas is partly innervated by the right vagus nerve by means of the hepatic and celiac branches. It may be affected by increased activity in the right vagal brainstem nuclei. We studied the right anterolateral medulla oblongata for the presence of arterial compression in 15 consecutive patients operated upon for right-sided cranial nerve vascular compression syndromes. A preliminary retrospective study showed that all 15 patients with type 2 diabetes had arterial compression of the right anterolateral medulla oblongata and that some could be relieved of their high blood sugar.[30] A controlled prospective study was carried out.

The present study extends the above data regarding the cranial nerve and hypertension to include autonomic dysfunction (type 2 diabetes mellitus [DM]) as a result of arterial elongation and pulsatile compression of the right anterolateral medulla oblongata.

Metabolic syndrome, insulin resistance and type 2 diabetes are all related to neural loop feedback dysfunction. Multiple metabolic changes are seen in these syndromes. Recent work revealed that the neuropeptides, especially the kinins and their G-protein–coupled receptors (B1, B2), are up-regulated in the medullary regions of the human brainstem. These changes are seen in both hypertensive and diabetic specimens.[31] These data implicate a regulatory role for these proteins in autonomic, motor and cardiovascular centers in the human so afflicted.

In diabetic rats, glucose transporters (GLUT) and accompanying mRNA are increased in the medulla oblongata, especially in the area of the ninth cranial nerve level of that structure.[32] In diabetes-induced animals, the monoamines, especially norepinephrine and serotonin, are seen to increase as a function of the length of time for the process.[33,34] This data suggests the progressive nature of diabetes is related to the progressive changes in the regional concentrations of specific brain monoamines, especially norepinephrine.[35] Infusion of norepinephrine in the lateral hypothalamic area (LHA) increases insulin secretion via increased vagal activity. Microinjection of glucose in this region results in efferent discharges in the right vagus, especially the pancreatic branches.[36] We found that decompression of the area of the brainstem involved with the vagal innervation reduced the downstream action of this vagal overactivity.

Our preliminary report was a retrospective study in which we performed MVD of the medulla in 15 consecutive patients with type 2 DM. We found that we not only had to relieve the pulsation but also the distortion of the brainstem due to the compression. Therefore, we developed a device which when placed against the medulla decreases both arterial pulsation and distortion.[30]

## METHODS

Ten patients with type 2 DM which was steadily progressive volunteered for this study, which was approved by the Institutional Review Board (IRB) on the basis of our prior preliminary study and our minimal morbidity and rare mortality (0.01%) in over 6,000 of these operations. The major decision we made was to operate on persons with no other symptoms such as facial pain, which if relieved might confuse the result of the medullary decompression on the DM.

**Figure 1:**
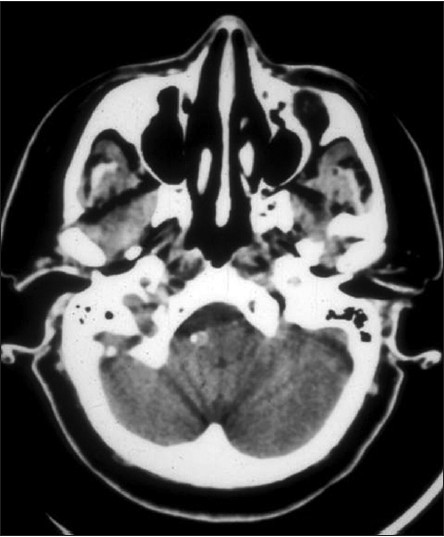
Patient number 3. MRI, sagittal view of medulla. Note right vertebral artery compressing anterolateral medulla

**Figure 2:**
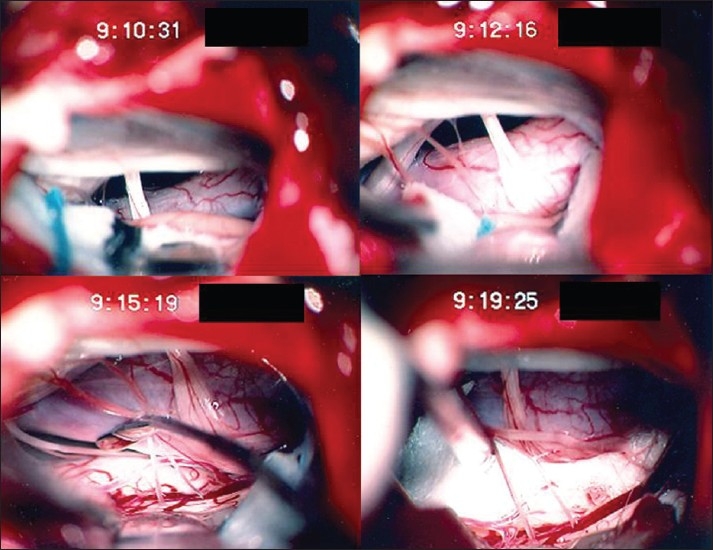
Patient number 3. Intraoperative photographs. Upper left — right vertebral artery (VA) compressing medulla. Upper right — VA being mobilized. Lower left — VA held off medulla. Lower right — implant of shredded Teflon felt and silicone strut (under felt and not seen) holding VA away from medulla

**Figure 3:**
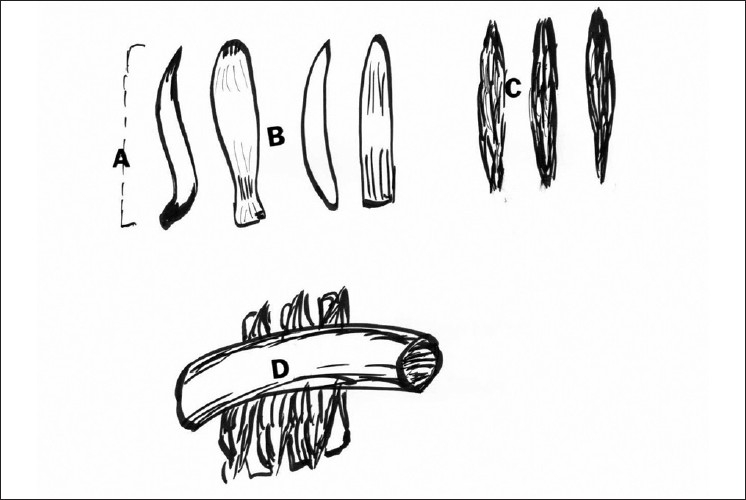
The double-layer vascular decompression device for relief of brainstem vascular grooving. Legend: a: 2-cm length; b: the carapace, curved silicone; c: the damper, PEFT shredded felt; d: the artery immobilized and decompressed using the carapace over the brainstem and the damper between the carapace and the artery

Ten patients with type 2 DM who were still making insulin, verified by C-peptide measurements, and had visible right lateral medullary compression by arterial loops on MRI scan underwent right retromastoid craniectomy and microvascular decompression of the medulla using previously described techniques of exposure (5, 6, 9, 21) and an innovative, patented implant [Figures 1–3]. A small (2.0 × 2.5 cm) low lateral retromastoid craniectomy was performed with the patient in the contralateral lateral decubitus position. The head was supported with a three-point head holder. After opening and retraction of the dura using stay sutures, the cerebellum was gently supported with a microsurgical retractor over a rubber dam and cottonoid. The medulla and cranial nerves X and XI were explored. The arterial loop was mobilized and then held away from the neural tissue using the double implant. Patients were followed for 12 months postoperatively. No changes in diet, weight or activity level were permitted during the first 12 postoperative months of the project. An addendum to the study protocol was approved by the IRB for follow-up to continue for another 12 months. However, not all patients agreed to participate in the extra 12 months.

The presence or absence and degree of arterial compression at operation were evaluated and recorded independently by the principal investigator and independently by neurosurgical colleagues.

The patients, 9 men and 1 woman, ranged in age from 43 to 63 years (mean age, 52.9 years) at the time of operation. Known duration of DM ranged from 1 to 20 years (mean, 6.8 years).

## MRI GRADING

MRI grading was performed preoperatively and postoperatively. Postoperative scans were performed 1 year postoperatively. The preoperative result was blinded at the time of the postoperative grading.

The scale used for grading was based upon which artery was compressing, designated by Roman numerals I-IV; and the amount of compression by the artery, designated by A-D, and O equaling ‘no artery adjacent to medulla’ — type I: vertebral artery (VA); II: posterior inferior cerebellar artery (PICA); III: both VA and PICA; IV: other; and compression grading O: no artery adjacent to medulla; A: artery proximate to medulla; B: artery mildly compressing medulla; C: artery moderately compressing medulla; D: artery severely compressing medulla.

## 2-HOUR POSTPRANDIAL GLUCOSE

Two-hour postprandial (2-hr pp) blood glucose levels (meal study) were measured preoperatively while patients were taking their diabetes medications, and also after they had gone through a ‘washout’ period of 1 week while not taking their diabetes medications. The first 3 subjects did not have the preoperative testing done with medications as this test was added to the protocol after they had been operated upon. Follow-ups of the meal study with medications were done at 3 and 6 months postoperatively. The meal study without medications was repeated at 12 months postoperatively.

The meal study that was done after a 1-week washout period of all diabetes medications was completed by all 10 participants preoperatively and 12 months postoperatively. Patients were admitted to the clinical research unit the night before their testing, where they received a balanced meal for dinner, based on their BMI, and then nothing by mouth after midnight. A radioactive glucose isotope 3-3H-glucose was infused intravenously for 3.5 hours (210 minutes) through “0” time. Standardized liquid breakfast of a commercial product, 7 kcal/kg, was consumed at 0 time. Blood samples were taken from an intravenous line (in the arm opposite the one that was used for the isotope infusion) every 15 minutes, starting at 30 minutes before ingestion of liquid meal through 120 minutes after ingestion. Samples were then taken at 30-minute intervals for the following 2 hours.

At the preoperative, 3-month, 6-month and 12-month meal studies, serum insulin, Hemoglobin (A_1_c), weight and medication audits were performed. Patients were instructed to make no changes in diet or exercise during the study period.

Plasma glucose levels were measured with the use of the YSI 2300 STAT glucose analyzer (Yellow Springs Instruments). Serum insulin levels were measured with the use of Immulite 2000 (DPC Diagnostic Products, Corp.) Hemoglobin A_1_c levels were measured using Bio-rad variant 21 and Phospholipase C (PLC).

Normal nondiabetic levels that were used for the metabolic assessments: FPG: 70-110 mg/dL; A_1_c: 4.0%-6.4%; serum insulin: <25 *μ*U/mL (microunits per milliliter); two-hour postprandial plasma glucose: <140 mg/dL.

## RESULTS

**Graph 1:**
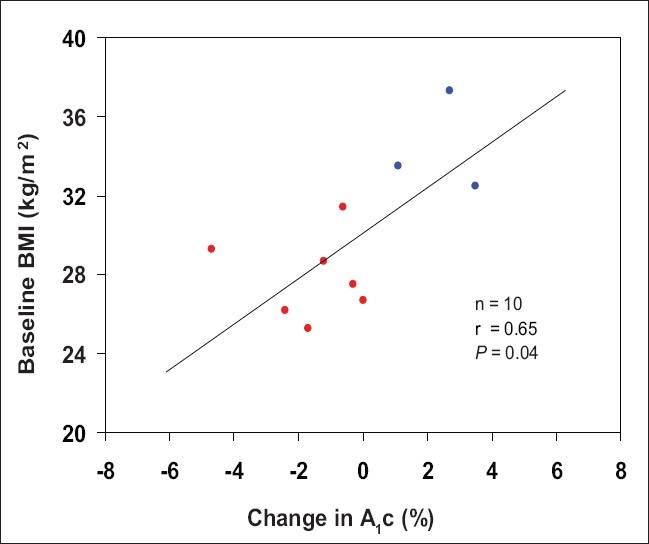
Relation between baseline BMI and changes in A_1_c

**Graph 2:**
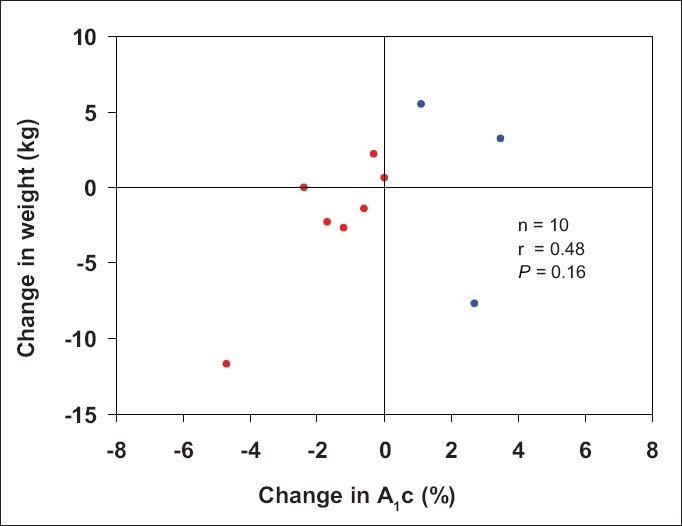
Relation between changes in weight and A_1_c

Results for individual lab, weight and medication measurements have been segregated into two different groups of ‘Good Responders’ (*n*= 7) and ‘Failed Responders’ (*n*= 3). Good responders‘ inclusion criteria required that overall response with regard to glycemic control either improved or did not worsen at the 12-month postoperative follow-up. Failed responders had no slowing in the natural progression of diabetes [Tables 1–5 Graphs 1, 2].

**Table 1:** MRI grading

1Subject#	Preop. MRI compression	OR compression	Postop. MRI compression
1	II C (III C)*	III C	II A/(B) (/B–implant?)
2	III B	IV C	I A/(B)
3	III C	III C	I A/(B)
4	I C	I D	0
5	I C	I D	0
6	IV B	IV C	0
7	I C	III C	II A
8	III B	III C	I A/(B)
9	III B	III D	I C/(D) (? implant)
10	I B	IV C	0

* Grading at second evaluation. Vascular compression grading-Type I: Vertebral artery (VA); Type II: Posterior inferior cerebellar artery (PICA); Type III: Both VA and PICA; Type IV: Other; O: No artery adjacent to medulla; A: Artery proximate to medulla; B: Artery mildly compressing medulla; C: Artery moderately compressing medulla; D: Artery severely compressing medulla; MRI: Magnetic resonance imaging

**Table 2:** Good-fail A1c < 7%

Good responders (n=7)							
Subject #	Preop.	3-mo F/U	6-mo F/U	12-mo F/U	Last F/U	Mos last F/U	Difference
1	8.3	7.9	8.4	7.1	6.9	56	1.2
2	6.2	5.7	4.6	5.6	6.8	51	0.6
3	9.6	7.6	7.4	9.3	8.6	56	1
4	10.1	7.6	7.8	7.7			2.4
5	8.1	6.2	6.1	6.4	6.6	45	1.7
6	10.8	6.1	5.6	6.1	7.2	48	4.7
9	7.5	9.3	7.3	7.5			0
Mean	8.657143	7.2	6.742857	7.1	7.22	51.2	1.657143
Rnd mean	8.7	7.2	6.7	7.1			1.7
Median				7.1	6.9		
Failed responders (n=3)							
7	7.4	7.0	7.8	8.5			
8	7.5	8.5	7.9	11.0	8.4	48	
10	10.6	12.1	12.5	13.3			
Mean	8.5	9.2	9.4	10.93333	8.4	48	
Rnd mean	8.5	9.2	9.4	10.9	8.4	48	
Total responders (n=10)							
Mean	8.61	7.8	7.54	8.25			
Rnd mean	8.6	7.8	7.5	8.3			

**Table 3:** Fasting blood glucose

Good responders (n=7)				
Subject #	Preop.	12-mo F/U	Last F/U	Mos last F/U
#1	132	146	116	56
#2	228	175	158	55
#3	196	242	170	57
#4	173	148		
#5	220	162	129	45
#6	190	110	137	48
#9	138	124		
Mean	182.4286	158.1429	142	52.2
Rnd mean	182	158		
Median	190	148	137	
Failed responders (n=3)				
#7	155	210		
#8	154	259	141	48
#10	308	273	187	45
Mean	205.6667	247.3333	164	46.5
Rnd mean	206	247		
Total responders (n=10)				
Mean	189.4	184.9		
Rnd mean	189	185		
Total responders (n=7)				
Mean			148.3	50.6
Rnd mean			148	

**Table 4:** Two-hour postprandial glucose

Good responders (n=7)						
2-hr PP	w/med Preop. #1	3-mo w/med F/U #1	6-mo w/med F/U #2	2-hr PP	no med Preop. #2	12-mo no med F/U #3
#1			273	#1	314	285
#2		268	293	#2	317	255
#3		293	281	#3	356	380
#4	333	313	402	#4	287	311
#5	399	243	279	#5	323	300.5
#6	335	295	253.5	#6	472.5	280.5
#9	249	251	258.5	#9	271.5	228.5
Mean	329	277.1667	291.4286	Mean	334.4286	291.5
Rnd mean	329	277	291	Rnd Mean	334	292
Failed responders (n=3)				2-hr PP		
#7	280.5	296.5	276.5	#7	147.5	363.5
#8	330.5	311.5	279.5	#8	350.5	478.5
#10	443	453	458	#10	466.5	460.5
Mean	351.3333	353.6667	338	Mean	321.5	434.1667
Rnd mean	351	354	338	Rnd Mean	322	434
Total responders (n=10)
Mean	338.5714	302.6667	305.4	Mean	330.55	334.3
Rnd mean	339	303	305	Rnd Mean	331	334

**Table 5:** Serum insulin

Good responders (n=7)				
	Preop.	3-mo F/U	6-mo F/U	12-mo F/U
#1	26.7	21	25	
#2		23	28	23
#3		9	8	
#4	5.6	15	23	6
#5	14	10	13	5
#6		8	10	2
#9	19	16	14	7
Mean	16.325	14.57143	17.28571	8.6
Rnd mean	16.3	14.6	17.3	9
Failed responders (n=3)				
#7		20	26	11
#8		10		9
#10	16	11		
Mean	16	13.66667	26	10
Rnd mean	16	13.7	26	10

### MRI grading

MRI findings in 10 patients are tabulated in Table 1. At intraoperative visual evaluation, the compression-distortion was more severe than seen on MRI scans in 9 of the 10 patients. The compression-distortion severity was the same on scan and intraoperatively in subject #3. MRIs performed postoperatively revealed improvement in the amount of compression-distortion in 9 of the 10 patients. Four of the patients had no visible compression, while 5 patients showed only mild distortion attributed to the device used at operation. The postoperative MRI of subject #9 demonstrated more compression-distortion than the preoperative MRI. We are unsure if this was actually due to the implant.

### Blood chemistry

The changes in hemoglobin A_1_c (A_1_c) following MVD are collated in Table 2. A_1_c was improved at 12 months in 7 patients and worse in 3 patients. Change in weight was minimal and had no effect on change in A_1_c. A body mass index (BMI) over 32 correlated with failure to improve in 3 patients. Graphs 1 and 2 are scattergrams of BMI and weight with A_1_c changes.

Fasting blood glucose levels are collated in Table 3. In the group of good responders to MVD (7 patients), the median fasting blood glucose dropped from 190 preoperatively to 148 at 1 year. In the group of failed responders (3 patients), the median fasting glucose rose from 206 to 247.

Two-hour postprandial glucose (PPG) levels are collated in Table 4. The preoperative 2-hour PPG had only a modest decrease postoperatively in the group of good responders. The group of failed responders showed a severe increase (without medicine) at 12 months, implying progression of the diabetes.

Fasting serum insulin levels are collated in Table 5. The data show significant decrease in the group of good responders at 12 months. The serum insulin was also decreased in the group of failed responders. The reason for this improvement in patients who otherwise deteriorated is unclear. Perhaps there was a salubrious effect of the MVD even though the other parameters were indicative of failure.

### Body mass index

Baseline BMI of responders was 27.9 ± 2.1 SD, while that of nonresponders was 34.4 ± 2.5 SD. There was little-to-no change of BMI in the participants at 12 months postoperatively.

## CONCLUSION

Of most significance is the finding that the normal course of disease progression was slowed in the majority of the participants. The reduction of diabetes medicines is also significant because of the decrease of the side effects associated with these medications that the patients may have to deal with . It is important to note that these improvements were seen while subjects made no behavioral changes, i.e., no weight change, no changes in diet or exercise/ activity levels. In clinical settings, life behavior changes of diet and exercise would be recommended and encouraged as is the standard of care. Body mass index appears to be an important factor in the outcome of this study. Those who had the best outcome from the intervention had BMIs identified as ‘overweight’, while those who did not respond had BMIs in the obese category, viz., 30 or greater. The significance of this autonomic/ metabolic abnormality spreads beyond type 2 DM. The implications are made with some surety that a number of problems of aging are due to arterial compression of the brainstem, specifically the medulla oblongata. The first of these problems is so-called essential hypertension, well known to neurosurgeons but with only early penetration into the general medical literature.

Many studies have been performed in attempts to show a genetic contribution in the inheritance of hypertension and diabetes. None of these have ever been able to complete the syllogism. It appears to us that one does not inherit hypertension. One does not inherit diabetes. Rather, one inherits blood vessels: veins and, especially, arteries. We inherit the size, the location, the proclivity for deterioration and elongation of arteries. We may possibly inherit the sensitivity of our myelin to pulsatile vascular compression.

As the senior author worked his way through the entities described above, caused by vascular compression of the cranial nerves and the brainstem, he found in the literature that many investigators had confused mechanism with etiology. The many excellent studies done on mechanism in diabetes are not mutually exclusive from the etiologic factors found here; rather they are complementary to this work.

Type 2 diabetes mellitus is associated with arterial pulsatile compression of the right anterolateral medulla oblongata, which appears to be an important etiologic factor. This etiologic factor must not be confused with mechanism. Instead, the many studies of the mechanism of type 2 DM correlate with, and complement this, etiologic process. Microvascular decompression may be an effective treatment for non-obese type 2 diabetes patients.

Source of Support: Highmark Blue Cross Blue Shield of Western Pennsylvania provided funding for laboratory and operative costs for this study.

## References

[1] Barker FG, Jannetta PJ, Bissonette DJ, Larkins MV, Jho HD (1996). The long-term outcome of microvascular decompression for trigeminal neuralgia. N Engl J Med.

[2] Barker FG, Jannetta PJ, Bissonette DJ, Shields PT, Larkins MV, Jho HD (1995). Microvascular decompression for hemifacial spasm. J Neurosurg.

[3] Benarroch EE (1994). Neuropeptides in the sympathetic system: Presence, plasticity, modulation, and implications. Ann Neurol.

[4] Chen CC, Yang JC (1991). Effects of short and long-lasting diabetes mellitus on mouse brain monoamines. Brain Res.

[5] de Sousa Buck H, Ongali B, Thibault G, Lindsey CJ, Couture R (2002). Autoradiographic detection of kinin receptors in the human medulla of control, hypertensive, and diabetic donors. Can J Physiol Pharmacol.

[6] Geiger H, Naraghi R, Schobel HP, Frank H, Sterzel RB, Fahlbusch R (1998). Decrease of blood pressure by ventrolateral medullary decompression in essential hypertension. Lancet.

[7] Jannetta PJ, Abbasy M, Maroon JC, Ramos FM, Albin MS (1977). Etilogoy and definitve microsurgical treatment of hemifacial spasm.Operative techniques and results in forty-seven patients. J Neurosurg.

[8] Jannetta PJ, Gendell HM (1979). Clinical observations on etiology of essential hypertension. Surg Forum.

[9] Jannetta PJ, Hollihan L (2004). Type 2 diabetes mellitus, etiology and possible treatment: Preliminary report. Surg Neurol.

[10] Jannetta PJ, Levy EI, Clyde B, McLaughlin MR (1998). Medullary compression and hypertension. J Neurosurg.

[11] Jannetta PJ, Moller MB, Moller AR (1984). Disabling positional vertigo. N Engl J Med.

[12] Jannetta PJ, Segal R, Wolfson SK, Dujovny M, Semba A, Cook EE (1985). Neurogenic hypertension: Etiology and surgical treatment. II. Observations in an experimental nonhuman primate model. Ann Surg.

[13] Jannetta PJ, Segal R, Wolfson SK (1985). Neurogenic hypertension: Etiology and surgical treatment. I. Observations in 53 patients. Ann Surg.

[14] Jannetta PJ (1970). Electromyographic and electron microscopic correlates in hemifacial spams treated by microsurgical relief of neurovascular compression. Surg Forum.

[15] Jannetta PJ (1979). Microsurgery of cranial nerve cross-compression. Clin Neurosurg.

[16] Jannetta PJ (1970). Microsurgical exploration and decompression of the facial nerve in hemifacial spasm. Curr Top Surg Res.

[17] Jannetta PJ (1975). Neurovascular cross-compression in patients with hyperactive dysfunction symptoms of the eighth cranial nerve. Surg Forum.

[18] Jannetta PJ (1977). Observations on the etiology of trigeminal neuralgia, hemifacial spasm, acoustic nerve dysfunction and glossopharyngeal neuralgia. Definitive microsurgical treatment and results in 117 patients. Neurochirurgia (Stuttg).

[19] Jannetta PJ (1967). Structural mechanisms of trigeminal neuralgia. Arterial compression of the trigeminal nerve at the pons in patients with trigeminal neuralgia. J Neurosurg.

[20] Jannetta PJ (1975). The cause of hemifacial spasm: Definitive microsurgical treatment at the brainstem in 31 patients. Trans Sect Otolaryngol Am Acad Ophthalmol Otolaryngol.

[21] Jannetta PJ (1977). Treatment of trigeminal neuralgia by suboccipital and transtentorial cranial operations. Clin Neurosurg.

[22] Jannetta PJ (1975). Trigeminal neuralgia and hemifacial spasm--etiology and definitive treatment. Trans Am Neurol Assoc.

[23] Jho HD, Jannetta PJ (1995). Microvascular decompression for spasmodic torticollis. Acta Neurochir (Wien).

[24] Kobayashi M, Nikami H, Morimatsu M, Saito M (1996). Expression and localization of insulin-regulatable glucose transporter (GLUT4) in rat brain. Neurosci Lett.

[25] Lacković Z, Salković M, Kuci Z, Relja M (1990). Effect of long-lasting diabetes mellitus on rat and human brain monoamines. J Neurochem.

[26] Laha RK, Jannetta PJ (1977). Glossopharyngeal neuralgia. J Neurosurg.

[27] Levy EI, Scarrow AM, Jannetta PJ (2001). Microvascular decompression in the treatment of hypertension: Review and update. Surg Neurol.

[28] Møller MB, Møller AR, Jannetta PJ, Jho HD, Sekhar LN (1993). Microvascular decompression of the eighth nerve in patients with disabling positional vertigo: Selection criteria and operative results in 207 patients. Acta Neurochir (Wien).

[29] Møller MB, Møller AR, Jannetta PJ, Jho HD (1993). Vascular decompression surgery for severe tinnitus: Selection criteria and results. Laryngoscope.

[30] Morimoto S, Sasaki S, Takeda K, Furuya S, Naruse S, Matsumoto K (1999). Decreases in blood pressure and sympathetic nerve activity by microvascular decompression of the rostral ventrolateral medulla in essential hypertension. Stroke.

[31] Naraghi R, Gaab MR, Walter GF, Kleineberg B (1992). Arterial hypertension and neurovascular compression at the ventrolateral medulla. A comparative microanatomical and pathological study. J Neurosurg.

[32] Naraghi R, Geiger H, Crnac J, Huk W, Fahlbusch R, Engels G (1994). Posterior fossa neurovascular anomalies in essential hypertension. Lancet.

[33] Niijima A, Kannan H, Yamashita H (1988). Neural control of blood glucose homeostasis; effect of microinjection of glucose into hypothalamic nuclei on efferent activity of pancreatic branch of vagus nerve in the rat. Brain Res Bull.

[34] Resnick DK, Jannetta PJ, Bissonnette D, Jho HD, Lanzino G (1995). Microvascular decompression for glossopharyngeal neuralgia. Neurosurgery.

[35] Segal R, Gendell HM, Canfield D, Dujovny M, Jannetta PJ (1979). Cardiovascular response to pulsatile pressure applied to ventrolateral medulla. Surg Forum.

[36] Yamamoto I, Yamada S, Sato O (1991). Microvascular decompression for hypertension--clinical and experimental study. Neurol Med Chir (Tokyo).

